# Supplementary material for: Crosslinking by Click Chemistry of Hyaluronan Graft Copolymers Involving Resorcinol-Based Cinnamate Derivatives Leading to Gel-like Materials
Source: Gels. 2024 Nov 19;10(11):751. doi: 10.3390/gels10110751 (PMC11594237; doi:10.3390/gels10110751)
Supplement: Supplementary file 1 [file gels-10-00751-s001.zip › gels-3272816-supplementary.pdf]

Supplementary data for

Crosslinking by Click Chemistry of Hyaluronan Graft Copolymers Involving Resorcinol-Based Cinnamate Derivatives Leading to Gel-Like Materials

Mario Saletti,[a,#] Simone Pepi,[a,#] Marco Paolino,[a] Jacopo Venditti,[a] Germano Giuliani,[a] Claudia Bonechi,[a] Gemma Leone,[a] Agnese Magnani,[a] Claudio Rossi,[a] Andrea Cappelli.[a,*]

[a]Dipartimento di Biotecnologie, Chimica e Farmacia, Università degli Studi di Siena, Via Aldo Moro 2, 53100 Siena, Italy.

# Mario Saletti and Simone Pepi contributed equally to the paper.

* Correspondence: andrea.cappelli@unisi.it.

Contents

Elaboration and applications of the technology platform employing **HA-FA-Pg** graft copolymers page 3

^{1}H NMR spectrum of the crosslinked material **HA(270)-FA-TEGERA-CL-10** page 4

^{1}H NMR spectrum of the crosslinked material **HA(270)-FA-HEGERA-CL-10** page 5

Swelling kinetics of **HA(270)-FA-TEGERA-CL** and **HA(270)-FA-HEGERA-CL** series page 6

Viscosity curves of **HA(270)-FA-TEGERA-CL** and **HA(270)-FA-HEGERA-CL** series page 7

Weight loss (%) of **HA(270)-FA-TEGERA-CL** and **HA(270)-FA-HEGERA-CL** series page 8

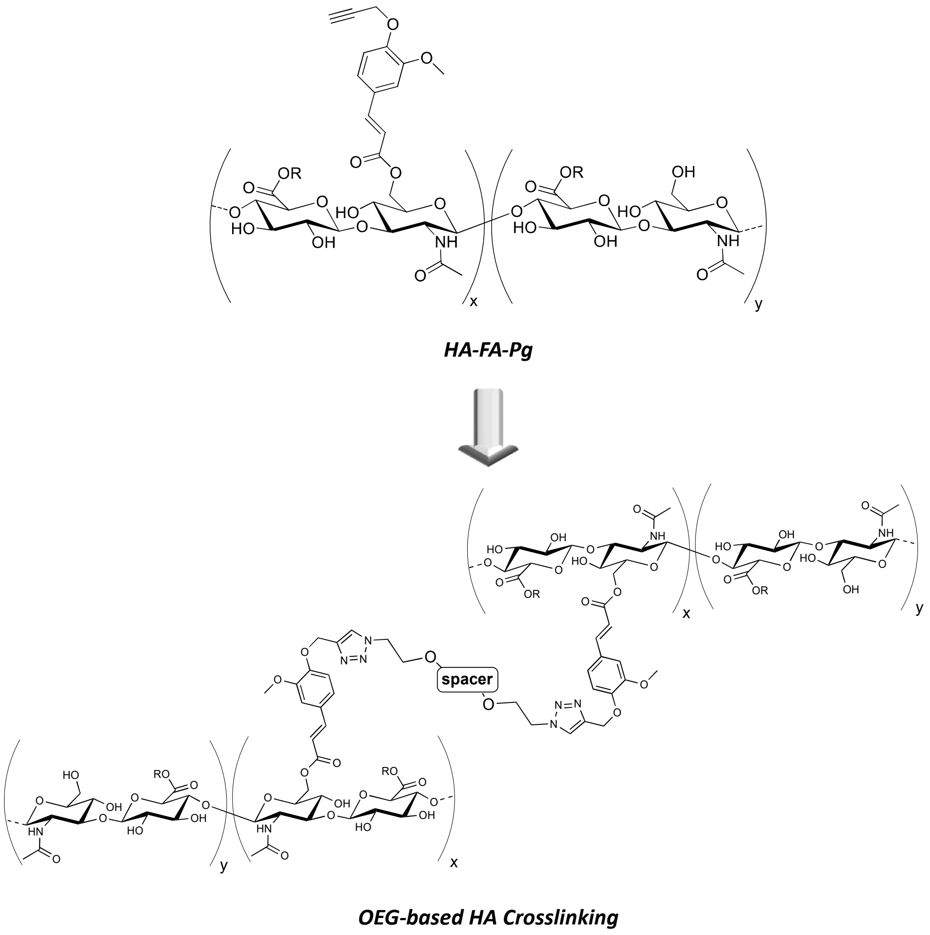

Figure S1. Elaboration and applications of the technology platform employing **HA** by means of **HA-FA-Pg** graft copolymers in OEG-based **HA** crosslinking.

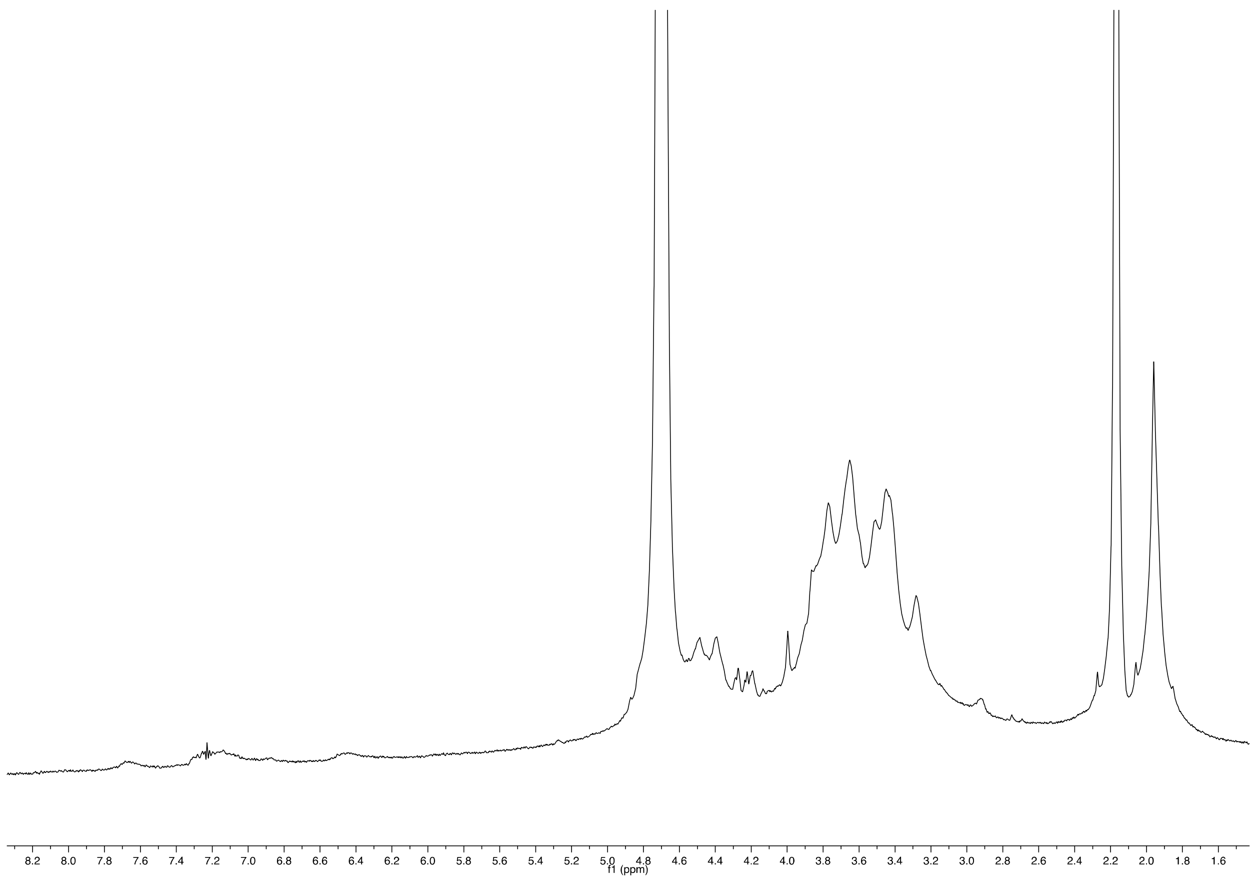

Figure S2. ^{1}H NMR (D_2O, 600 MHz) spectrum of the crosslinked material **HA(270)-FA-TEGERA-CL-10**.

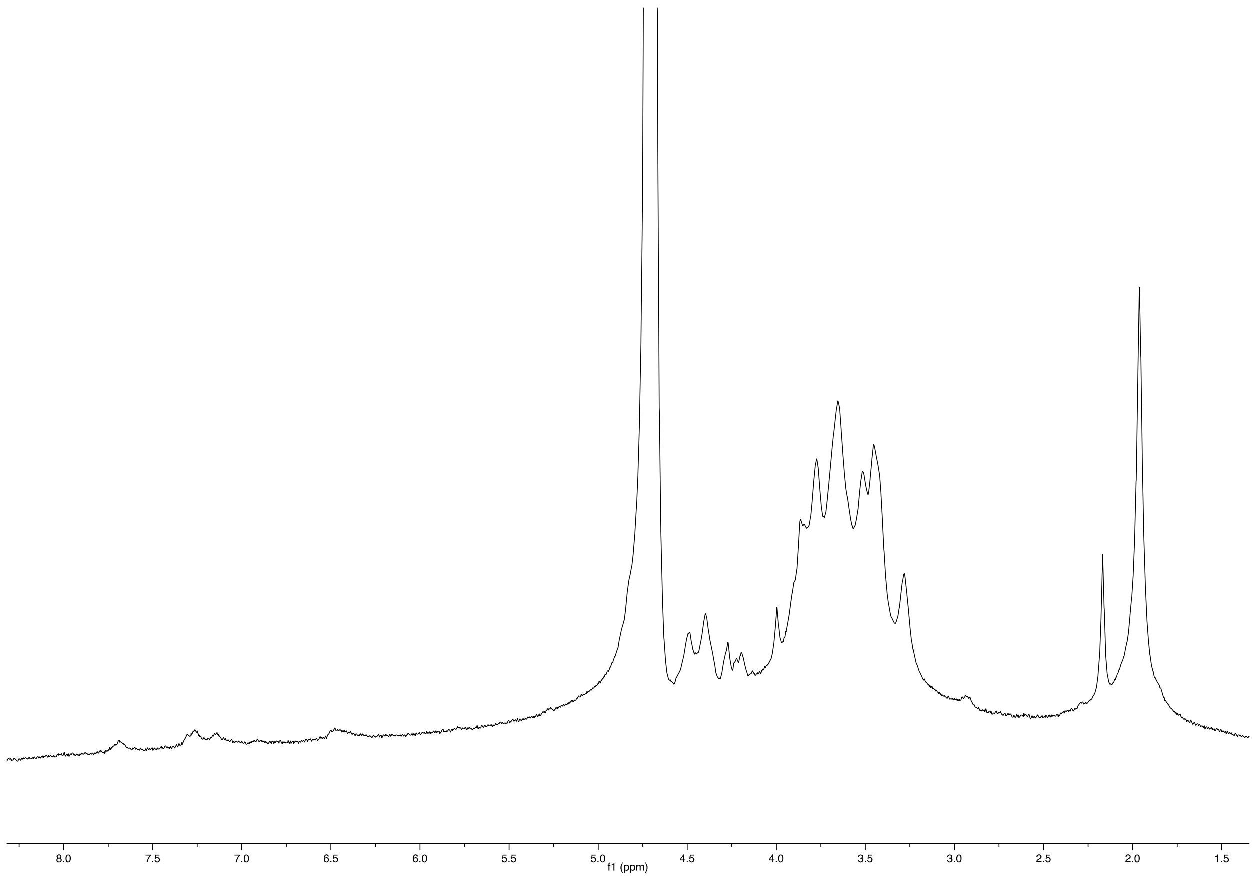

Figure S3. ^{1}H NMR (D_2O, 600 MHz) spectrum of the crosslinked material **HA(270)-FA-HEGERA-CL-10**.

(A)

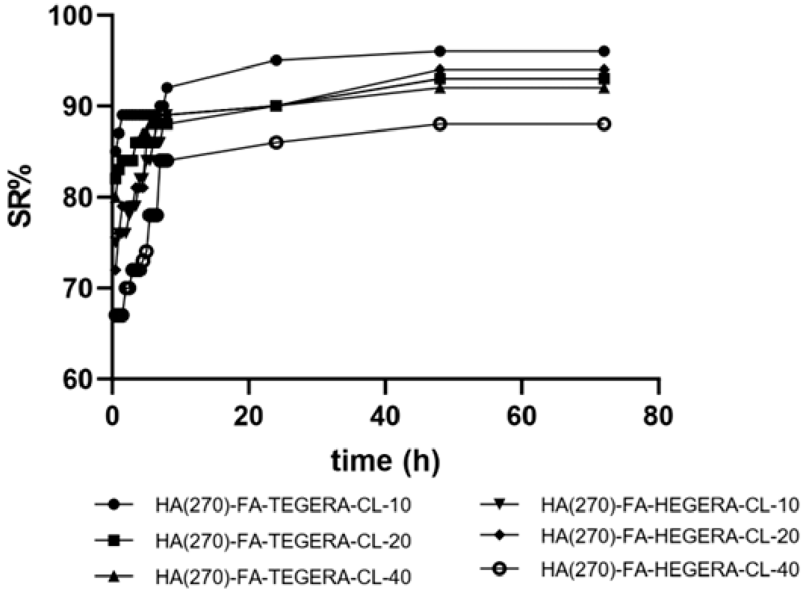

(B)

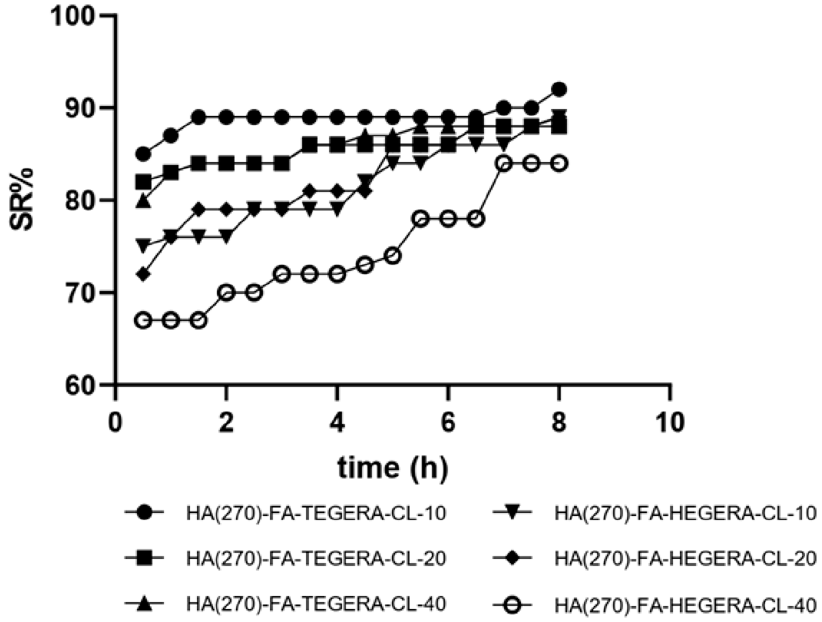

Figure S4. (A) swelling kinetics measured across the time range 0-72 h of **HA(270)-FA-TEGERA-CL** and **HA(270)-FA-HEGERA-CL** series. (B) swelling kinetics measured across the time range 0-8 h of **HA(270)-FA-TEGERA-CL** and **HA(270)-FA-HEGERA-CL** series.

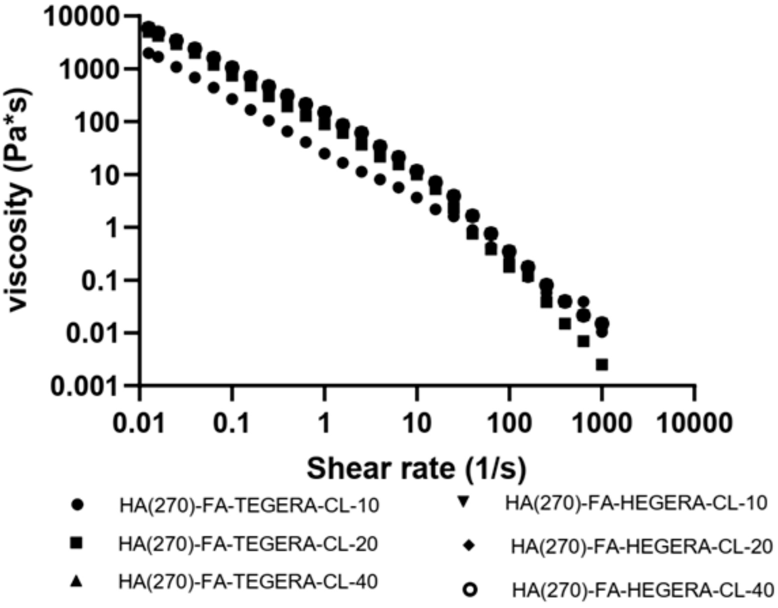

Figure S5. Viscosity curves of **HA(270)-FA-TEGERA-CL** and **HA(270)-FA-HEGERA-CL** series.

Table S1. TGA analysis of samples in the 30-850 °C heating range. Data were reported as % mean values (n = 3).

**Sample**	**Heating range 30-850 °C**
**HA(270)-FA-TEGERA-CL-10**	80 ± 1%
**HA(270)-FA-TEGERA-CL-20**	88 ± 1%
**HA(270)-FA-TEGERA-CL-40**	78 ± 2%
**HA(270)-FA-HEGERA-CL-10**	81 ± 1%
**HA(270)-FA-HEGERA-CL-20**	81 ± 1%
**HA(270)-FA-HEGERA-CL-40**	82 ± 1%
**HA(270)-FA-HEG-CL-10**	86 ± 2%
**HA(270)-FA-HEG-CL-20**	89 ± 1%
**HA(270)-FA-HEG-CL-40**	91 ± 1%
**HA(270)-FA-Pg-10**	92 ± 1%
**HA(270)-FA-Pg-20**	91 ± 1%
**HA(270)-FA-Pg-40**	91 ± 1%
